# 1896: A Retrospect

**Published:** 1897-01-02

**Authors:** 


### 1896: A Retrospect.

So far as progress is to be measured by the introduction of novelties, new modes of practice, new methods of diagnosis, and new discoveries in pathology, it cannot be said that, with one exception, any great or striking advance has been made during the year 1896. Nevertheless, no one who has watched the course of events can have failed to see that great progress of an even surer sort has been made—that knowledge has been consolidated, that much that was but speculation has become part and parcel of daily practice, and that by the accumulation and comparison of experience by men engaged in special branches of professional work great advances have been made towards accuracy and certainty in the diagnosis of diseased conditions, in the proper selection of cases for operation, and in the technical details of the operations themselves.

### The Roentgen Rays.

The great novelty of the year has undoubtedly been the discovery by Roentgen of the form of energy which he has wisely described under the title of the *x* rays. The nature of these rays is as yet undecided, while the range of their utility is as yet unknown. Whether they are the result of longitudinal vibrations of ether, as opposed to the transverse vibrations by which ordinary light is supposed to be transmitted; whether they arise from vibrations comparable to those of ordinary light, but taking place at an infinitely greater velocity; or whether they are due to an actual material emission, seems as yet not to be established. Their use in the investigation of many surgical problems is, however, a matter of certainty. Never, perhaps, was any invention so immediately appropriated by the surgical profession, and at once put in harness for useful purposes as was this novel means of seeing into the dark places within the body. The presence of foreign bodies has been determined and their position exactly fixed by their means; the existence of calculi in the kidneys has been proved; the diagnosis between fracture and dislocation has been made clear in many obscure cases; the exact position of the bones in cases of fracture has been demonstrated; the lack of ossification in the ends of ricketty bones has been made obvious; and it has been possible to show the process of cure and the deposit of new bone within the softened structures. It has also been possible in some cases to map out certain of the internal organs, and to demonstrate the existence of tumours and even of aneurisms. Although the eye is quite insensible to these so-called *x* rays, it was soon found that certain salts became luminous when exposed to them, and thus they were in an indirect way made sensible to the retina, so that it is now possible to "see" the shadows cast by objects within the body without waiting for the production of a photograph. It must be said, however, that where accuracy is desired, the photographic picture gives the most reliable results, besides furnishing a permanent record of the appearance.

In our present state of ignorance as to the relation between this form of energy and ordinary light it is impossible to say how far its utility may in the future be extended, but there is some reason for thinking that, as at present produced, the *x* ray is a mixture of rays of various qualities, and that different substances are not equally opaque to all of them. Clearly, if this should turn out to be the case, and if it should be possible to produce the different qualities of rays separate from one another, it might be possible not merely, as at present, to point out the existence of a shadow, but to say with some approach to accuracy what is the nature of the substance by which such shadow is produced. At present what seems certainly known amounts to this—that the rays pass in direct lines through all the substances which they are able to traverse, that the pictures thus produced by them are only shadow prints and not true images, and that the opacity of different substances to the rays varies roughly according to their specific gravity, so that as a rule a dark patch in the skiagraph, as the pictures are now called, represents a substance of greater density than the surrounding media—bones are darker than muscles, stones and pieces of glass show up darkly among the tissues amid which they have become impacted, and, of all substances, bullets, needles, and coins are most clearly rendered visible. The practical utility of all this, especially to the surgeon, is obvious, and the constant use to which the process of skiagraphy, a process which a year ago was utterly unknown, is now put in the daily work of the surgeon is but another proof of the receptivity of the medical profession of to-day, and of its readiness to assimilate knowledge from every quarter and to use it for the general good.

### The Diagnosis of Typhoid Fever.

Another novelty of far less importance, probably, but still one which, from the rapidity with which its practical application has been brought into daily use, shows the speed with which inventions are now passed through the sieve of experience, is the process for the diagnosis of typhoid fever by the reaction of typhoid blood upon a culture of the typhoid organism. Much has yet to be determined in regard to this, and perhaps it would be wrong to attribute too much importance to the discovery; nevertheless, it is another straw showing the direction of the wind, another fact pointing to the definiteness of the changes in the blood produced by specific fevers.

### The Treatment of Diphtheria.

The joint report of the superintendents of the hospitals of the Metropolitan Asylums Board was issued on March 28th. It contained statistics of the cases of diphtheria treated in 1895, during the whole of which year anti-toxic serum had been in use. The effect of this report was to confirm in every way the strong opinion formed by those who had used the serum in favour of its power in controlling the disease. By far the most striking results were obtained in the treatment of post-scarlatinal cases which occurred while the patients were under observation, which again went to confirm the view that the earlier the serum was used the better would be the results.

Since then, however, great advances have been made in the production of diphtheria anti-toxin, both in regard to concentration and to definiteness of strength. The serum can now be supplied of a much greater intensity than formerly was attainable, and steps have been taken to materially reduce the price of these concentrated forms, which has hitherto been prohibitive. The advantage of obtaining an anti-toxin of greater intensity is very considerable, for all evidence points to the desirability of giving a large dose at first, so soon as the disease is recognised, and the injection of the large quantity of the weaker serum required to carry the desired quantity of anti-toxin is in various ways objectionable.

### Serum Treatment of Septic Diseases.

A step forward has been made in regard to the treatment of septic diseases, and especially puerperal fever, by anti-streptococcic serum. A considerable number of cases have been reported in which benefit has appeared to follow the treatment. In other cases, however, the result has been disappointing, but in regard to this it must be borne in mind how often septic diseases are due to a mixed infection by a variety of micro-organisms. It would be contrary to all we know on the subject of the action of anti-toxins to expect such diseases, arising from such a variety of toxins, to be cured by a serum containing the anti-toxin related to one form of micro-organism alone. There are several sources of fallacy in regard to the use of serum in the treatment of diseases which appear to be due to septic organisms, but especially is it to be remembered that in many cases where streptococci undoubtedly are present they may not be the primary or even the main cause of the disease, and that, where this is the case, the attempt to cure by the use of the anti-toxin for the streptococcus would be no more likely to succeed than it would be if used for the cure of diphtheria in which, as is well known, streptococci are sometimes present in such large numbers as to have led observers in the earlier days of bacteriology to look upon them as the real cause of the disease.

### The Malaria Parasite.

The etiology of malaria has been a good deal discussed during the year, and, although it cannot be said that we have learned anything very new in regard to it, such knowledge as we possessed has undoubtedly been brought to a point and made much clearer, and the gaps in our knowledge have been indicated with much precision in the admirable Gulstonian lectures delivered by Dr. Patrick Manson. He has especially concerned himself in showing (1) that the malaria parasite can not only exist outside the body quite independently of man, but that it can go on multiplying there indefinitely. As he says, we know that malaria abounds in tropical wildernesses where man is never seen, therefore we are forced to assume (2) that man is in no way necessary to its existence. Is, then, the intrusion of the parasite into man a mere accident? To this he answers emphatically, No! and says (3) that man must be looked on as an alternative host. But that being so, it becomes necessary to assume that, as provision is made for the entry of the parasite into the body, so provision must be made for its exit, and (4) such provision, he maintains, can be found in the operations of certain suctorial insects common in the hot, damp regions where malaria prevails—the mosquito theory. What Dr. Manson believes to be the life history of the malaria parasite, then, is shortly this: it is really a parasite of the mosquito, passing from one individual and one generation of mosquito to another, owing to the fact that the larvæ of this insect devour the dead bodies of their parents, and thus in turn become infested with their parasites; but when swallowed by man in water, as so many disease germs are, or when breathed, from the ponds drying up and the plasmodia in the larvæ, or those scattered about in the water, passing into a resting stage and rising in the air in the form of dust, it may find its way back to the human host from whom its ancestors had started many generations back. These lectures have given rise to a considerable amount of discussion and of criticism.

### The Royal Commission on Tuberculosis.

The Commission upon Tuberculous Milk and Meat has confirmed and approved every position taken up by the scientific experts who gave evidence before it. Tuberculosis has been demonstrated to the world to be an infectious disease, communicable by milk and meat, and yet preventable by elementary sanitary measures which the least learned can understand and carry out in practice. This demonstration has been made once for all; and it may be many years before it bears all the fruit it is capable of bearing.

### The Treatment of Heart Disease.

Among the medical topics which have been much discussed during the year a place must be found for the treatment of heart disease by baths and exercises, more especially as it is carried out at Nauheim. It is easy to accept the fact that many cases of extreme heart failure and rapidly extending dilatation have done well under this treatment without admitting all that has been advanced in explanation of the good that has resulted. The treatment has been a frequent subject of discussion in the journals and medical societies, and was the subject of a full debate at the Carlisle meeting of the British Medical Association, where the discussion was introduced by Sir T. Grainger Stewart. Much has all along been made of the very remarkable alteration of the so-called size of the heart, which is stated to occur during each of the processes involved, namely, the baths and the exercises; and many diagrams have been exhibited, showing how great this has been and how quickly it has taken place. As to this also we would say that while we would not for a moment hesitate to accept the statements made as correct records of careful observations, we do not think that the profession will at once accept all the deductions which have been sought to be drawn from these observations, or will admit that of necessity they indicate a lessening of the size of the heart as great as they have by some been thought to show. In regard to this a careful paper by Dr. Watson Williams in the *British Medical Journal* of September 19th ought to have attracted more attention than it seems to have done. Dealing with the matter as a merely physical problem, he shows how little relation exists between alterations in the area of deep cardiac dulness and alteration of the actual diameter of the heart, a very small increase in the size of the heart affecting the resonance on percussion over a disproportionately large area of the chest wall. The considerations set forth in this paper seem to invalidate many of the observations which have been most confidently relied upon to prove the occurrence of rapid alteration in the size of the heart.

### Hospital Reforms.

The attention of the profession has been largely drawn at various times during the year to questions of medical ethics, medical defence, hospital abuse, the evils of provident dispensaries as at present conducted, and to various other matters having their root in the overcrowding of the profession and the difficulty of making a living in general practice. Many proposals have been made, but nothing very new has been done in regard to any of these grievances, except that an association has been started, under the name of the Hospital Reform Association, which has begun to collect information on the subject, and intends to make a vigorous effort to check some of the abuses which have crept into the system of free medical relief as seen in the out-patient departments of many of our large hospitals.

### Ethics and Advertising.

The question of medical advertising has been much discussed, sometimes with considerable acrimony. It cannot, however, be said that as a result either the laws or the customs of the profession have become any more strictly defined than they were before. Nor, in fact, are they likely to become so. It seems impossible to lay down rules capable of keeping out the sinner without excluding just men along with him; and the acrimonious wrangling which followed an attempt on the part of the *British Medical Journal* to suppress what was editorially considered an evil practice and a bad example in regard to certain door-plates or advertisements has only tended to show how difficult it is to draw any hard and fast line between right and wrong in such matters. Not that we would suggest for a moment that there is not a real difference between what is ethically correct and much that is obviously wrong; but when in practice any attempt is made to draw a definite line between the sheep and the goats difficulties arise, it soon ceases to be a question of broad principles, and we become engulfed in petty questions as to the size and position of a door-plate, the character of the newspaper in which one's books are advertised, the action of a hospital secretary in publishing the names and addresses of the staff, or the action of an insurance company in printing the names of its medical examiners, and those who have been tempted to try their hands at drawing a dividing line between what is permissible and what is wrong have too often found to their sorrow that they have undertaken an impossible task. In this, as in so many other things, it must be the general voice of professional opinion which must decide in each individual case, and must exercise pressure upon each individual waverer. This is the teaching of the events of the year in regard to ethics. It is nothing new—is old as the hills, in fact —but it is none the less worthy of consideration.

### The Seal of Secrecy.

On one subject the events of the year have certainly tended to enforce old rules and to draw attention to old and honourable traditions as to professional conduct which seemed by many to have almost been forgotten. As an abstract principle, the old rule of professional secrecy in regard to anything learned during professional intercourse has never been either forgotten or denied. Nevertheless, the extremely varied opinions which were expressed, even by leaders in the profession, on the occasion of a well-known trial, were sufficient to show how dulled the professional conscience had become on this question, and how ready it was to be taken in by the petty sophistries of individual conundrums, instead of sticking to the one guiding principle of maintaining a secrecy, equal at least to that of the confessional, in regard to professional confidences. The widespread laxity which came to light on that occasion was, no doubt, a shock to those who had thought upon the subject. The probabilities are that a great many medical men had really never thought seriously on the subject at all. But now they have there is every reason to hope that the outcome of the discussion which arose over this *cause célèbre* has been greatly to strengthen the professional conscience in regard to the sanctity of the revelations made to medical men in the course of their profesional duties. Conscience, however, is always the better for the sanction of law; and, although we have no doubt about the law on the subject, we certainly wish that we could have some assurance that all the judges would think alike in regard to the details of its application.

It is a sign of the times, and worthy of record, that Dr. H. St. Clair, in bringing an action against an accident insurance company for a sum due to him in consequence of disablement arising from an infected wound, submitted to an adverse verdict rather than disclose the name of the patient from whom he had received the infection. The Lord Justice Clerk said there was no reason to doubt the *bona fides* of the pursuer, who had acted in an honourable manner in not disclosing the name of the patient whom he was attending. But while that was so, his lordship was bound to arrive at the result that the pursuer had not proved his case. So he went without his money. But this is a case that shows that we still have among us men who are ready to maintain the right and the duty of reticence even at the expense of personal sacrifice.

In professional ethics the event of the year certainly has been the rehabilitation of the old obligation of professional secrecy.

### The Royal Commission on Vaccination.

The past year has in a manner been a year of jubilees and celebrations. In it we have celebrated the centenary of vaccination, the jubilee of anæsthesia, and, in a smaller way, the jubilee of the establishment of the Pathological Society.

It was but appropriate that in the year in which we celebrated the centenary of vaccination there should be issued the most complete proof and vindication of its efficacy that has yet been given to the world, viz., the Report of the Royal Commission on Vaccination. The ponderous volumes in which the evidence given before this Commission is contained form a well of information on the subject, and must at least be accepted as containing every argument that the wit of man has so far been able to devise against the proceeding, while the carefully drawn up Report of the Commission gives such a logical proof of the efficacy of vaccination, and of the evils which follow its neglect, that the reader of its arguments must wonder that they were not carried to their logical conclusion, that is, to the recommendation of compulsory vaccination and re-vaccination. Such, however, was not the case, and at present the position of affairs savours more of political compromise than of scientific deduction. It is to be hoped that the inquiry which Dr. Thorne Thorne is now conducting, as to the methods of vaccination by which certain Continental nations have been able to obtain the very complete protection from small-pox which they appear to enjoy, may eventuate in a Bill acceptable to scientists as well as to politicians.

The Jubilee of Anæsthesia.

As in regard to vaccination so in reference to anæsthetics, it is remarkable how long each process remained unchanged and unimproved. Vaccination was given to the world a hundred years ago in the same crude, unfinished form in which it is now practised, and for nearly a hundred years the protection given by it remained an isolated fact, an unexplained coincidence.

In the very earliest days of anæsthetics the substances used were nitrous oxide gas and ether, and almost within a year of the use of the latter in surgery chloroform was introduced; yet, to the present day, these three drugs, notwithstanding the many attempted modifications, remain the anæsthetics in daily use. Nor can it be said that much improvement has taken place in their mode of administration, beyond the merely mechanical arrangements in the case of gas and ether, which have been found necessary to prevent their undue dilution. As regards chloroform, many members of the profession are still in the dark ages, and, while the phrase "chloroform fatality" seems almost a standard headline in the medical journals, continue to administer the drug in such a manner as to use vastly larger doses than are known to be quite efficient for the purpose; doses, in fact, which would certainly be fatal if they were absorbed. In this respect 1896 has shown itself but slightly different from 1847. It is well known, however, that this need not be, and that the dosage of chloroform can be regulated as well as that of any other drug.

The Royal College of Physicians.

The Royal College of Physicians of London has during the year completed and published the "Nomenclature of Diseases," Dr. J. F. Payne being the editor of the volume.

Sir S. Wilks was appointed President by a large majority, and soon afterwards he had the honour of being appointed one of the Physicians in Ordinary to Her Majesty the Queen.

The Royal College of Surgeons.

The Royal College of Surgeons does not get much further in regard to matters of reform. By meetings and votes, and by circulars and by a general poll of the Fellows in regard to representation of Members on the Council, a good deal of steam has been blown off, but in view of the curious action of the Council in March last it does not appear likely that even the most vigorous expression of outside opinion will have much influence on their decision. What they did on that occasion was to firmly bury their heads in the sand, and, shutting their ears to all that had ever been said on the subject, to pass the following resolution: "That, as the members of this Council represent the body corporate of this Royal College, and consequently its Members as well as its Fellows, it is the opinion of this Council that no further representation of the Members is desirable?" After this, what can one expect?

Sir William MacCormac was elected President in July. We are glad to be able to state that he is now steadily recovering from the attack of pneumonia and empyæma, from which he has been suffering.

De Mortuis.

In no year since the birth, now ten years ago, of this journal have we been called upon to record the death of so large a number of men eminent in science and medicine as in 1896. The President of the College of Physicians, Sir Russell Reynolds, while hardly yet familiar with the high duties of his office, was called to lay down his authority. Of him it was said that no president ever more truly deserved, and held, the respect of his distinguished colleagues. No man grudged him the honour of his elevation, and no man but mourned his all too premature death. Within the year Sir George Johnson has died, a man who, to high scientific capacity and attainments, joined strenuousness, courage, and honesty of purpose in a very eminent degree. Sir John Erichsen, too, but at a riper age, has passed away. He was a man "born to much honour from his cradle." When, late in life—too late, indeed, for his merits—a baronetcy was conferred upon him, all his professional brethren felt that justice had at length been done. The University of Cambridge, and with her the whole medical profession, have to mourn the loss of Sir George Murray Humphry. Sir George Humphry, it is said, "created a medical school" at Cambridge. He "built" for himself, also, by his life and work in connection with that ancient University, "an everlasting name." Dr. George Harley also has to be numbered among those who are known no more among the living. His life was devoted to high purposes, and lived on a high personal and professional plane. We have space but to mention one name more, that of Sir Benjamin Ward Richardson. He was a frequent and valued contributor to this journal, and all his papers were marked by ingenuity, conspicuous honesty, and high scientific merit. Humanity and medicine are alike the poorer for his loss.

"The Hospital" to its Readers.

Such has been the year that has just now passed away. It may not figure largely in the making of medical history. It has, however, been full of events. Each year as it passes brings to the chronicler more and more to chronicle; each year as it comes to an end seems further and further from its beginning. The multitudinous events which crowd upon the stage as weeks and months go by push back the preceding January into the dim distance, when it is seen through the long vista of memories raised by all that has happened since it passed into history.

Every year the work of the medical journalist becomes more difficult, not because there is less to say, but because there is less time in which to listen, and the selection of material becomes so much the more important. The Hospital, however, will do its best, and will try to offer week by week to its readers a reflex of what is going on in those institutions, which are, and always must be, the centres of thought and progress in all that concerns the medical sciences. Another year has gone, and that we put behind us. Another year is opening, which will doubtless be as full of interest as any that have preceded it, but before we plunge into the task of chronicling its events we pause to wish good health, prosperity, and great happiness to all our readers.

